# SARS-CoV-2 Transmission to Masked and Unmasked Close Contacts of University Students with COVID-19 — St. Louis, Missouri, January–May 2021

**DOI:** 10.15585/mmwr.mm7036a3

**Published:** 2021-09-10

**Authors:** Terri Rebmann, Travis M. Loux, Lauren D. Arnold, Rachel Charney, Deborah Horton, Ashley Gomel

**Affiliations:** 1 Saint Louis University, St. Louis, Missouri.

Universities open for in-person instruction during the 2020–21 academic year implemented a range of prevention strategies to limit the transmission of SARS-CoV-2, the virus that causes COVID-19, including physical distancing, mask use, vaccination, contact tracing, case investigation, and quarantine protocols (*1*). However, in some academic programs, such as health-related programs, aviation, and kindergarten through grade 12 (K–12) education, maintaining physical distance while still providing instruction is difficult; for universities with such programs, a single confirmed case of COVID-19 could result in a large number of students, staff members, and instructors being designated close contacts and requiring quarantine if they are not fully vaccinated, even if masks were worn when contact occurred. In January 2021, the St. Louis City Health Department allowed Saint Louis University (SLU) to implement a modified quarantine protocol that considered mask use when determining which close contacts required quarantine.* To assess the impact of the protocol, SLU assessed positive SARS-CoV-2 test result rates by masking status of the persons with COVID-19 and their close contacts. During January–May 2021, 265 students received a positive SARS-CoV-2 test result; these students named 378 close contacts. Compared with close contacts whose exposure only occurred when both persons were masked (7.7%), close contacts with any unmasked exposure (32.4%) had higher adjusted odds ratios (aORs) of receiving a positive SARS-CoV-2 test result (aOR = 4.9; 95% confidence interval [CI] = 1.4–31.1). Any additional exposures were associated with a 40.0% increase in odds of a positive test result (aOR = 1.4; 95% CI = 1.2–1.6). These findings reinforce that universal masking and having fewer encounters in close contact with persons with COVID-19 prevents the spread of SARS-CoV-2 in a university setting. Universities opening for in-person instruction could consider taking mask use into account when determining which unvaccinated close contacts require quarantine if enforced testing protocols are in place. However, this study was conducted before the B.1.617.2 (Delta) variant became the dominant strain of SARS-CoV-2 in the United States, which could have affected these findings given that the Delta variant has been found to be associated with increased transmissibility compared to previous variants.

In January 2021, the St. Louis City Health Department allowed SLU to implement a modified quarantine protocol that considered mask use when determining who would be considered a close contact requiring quarantine. SLU is a mid-sized private university with approximately 12,000 students and 6,000 employees, approximately 80% of whom lived, worked, or studied on its urban campus during the spring 2021 semester (January–May 2021), when the modified policy was implemented. Mask use was enforced on campus for all students, staff members, and visitors, including outside and in all classrooms and laboratory spaces. Faculty and staff members asked those who were unmasked or improperly wearing a mask to comply. Noncompliant students received sanctions, including being unable to attend classes in-person. While actively eating or drinking, persons were allowed to be unmasked in dining halls. COVID-19 cases among students were identified through SLU’s symptomatic and surveillance testing protocols.^†^

After a case in a student was identified, the SLU Contact Tracing team conducted contact tracing. Staff member exposures and cases were tracked differently and were excluded from this study. Students with COVID-19 were asked to identify their number of exposure incidents with each close contact, defined as each single encounter in which the two persons were within 6 feet of each other for ≥15 minutes during a 24-hour period, regardless of location of the exposure. The number of exposures was then calculated for each close contact. Close contacts could have had a single exposure to one infected student or multiple exposures to one or more infected persons. Mask use for persons with COVID-19 and close contacts was recorded for each exposure incident. If either person (patient or contact) was unmasked, the incident was considered an unmasked exposure. All close contacts underwent saliva-based molecular reverse transcription–polymerase chain reaction (RT-PCR) testing 5–7 days after exposure.^§^ Only unvaccinated close contacts with unmasked exposures were required to quarantine; however, close contacts with only masked exposures were informed of their exposure to persons with COVID-19 and counseled to conduct daily health screenings and to immediately report any symptoms.

The percentages of positive SARS-CoV-2 test results among close contacts were assessed by demographic characteristics and exposure variables; differences were tested using chi-square tests or Fisher’s exact test for small cell counts for categorical variables and using *t*-tests for continuous variables; p-values <0.05 were considered statistically significant. Assessed demographic characteristics included sex, age, on- versus off-campus residence, vaccination status, enrollment as an undergraduate student versus graduate student, participation in school athletics, and membership in a student fraternity or sorority, Reserve Officers’ Training Corps, or a health major with clinical responsibilities. Full vaccination was defined as having received either a single dose of Janssen (Johnson & Johnson) vaccine or the second dose of Moderna or Pfizer-BioNTech COVID-19 vaccine ≥14 days before exposure. Partial vaccination was defined as receipt of a single dose of Janssen or the second dose of Moderna or Pfizer-BioNTech <14 days before the exposure, or receipt of 1 dose of Moderna or Pfizer-BioNTech COVID-19 vaccine. Students who had received no vaccine doses were considered unvaccinated. Close contacts were categorized as those who had only masked exposure or those with unmasked exposure to a COVID-19–infected person. Logistic regression was used to calculate adjusted odds ratios and 95% CIs pertaining to the odds of a close contact receiving a positive test result following masked versus unmasked exposure to a person with COVID-19, adjusting only for the number of exposure incidents^¶^; vaccination status was not included in the model because of small sample sizes. Most students were not eligible for vaccination during the spring semester. Statistical analyses were conducted using R software (version 4.1.1; R Foundation). The SLU Institutional Review Board approved this study.

A total of 9,335 student SARS-CoV-2 tests were performed during January–May 2021, including 1,009 (10.8%) diagnostic tests and 8,326 (89.2%) surveillance tests; students might have been tested more than once. Of all tests conducted, 265 (2.8%) yielded a positive SARS-CoV-2 result; no student received two positive test results. Among students with a positive SARS-CoV-2 test result, 378 close contacts were named (mean = 1.4 close contacts per case), 26 (6.9%) of whom reported only masked exposure; 352 (93.1%) reported any unmasked exposure. Close contacts had a median of one exposure incident (range = one–16). Reported exposures occurred between roommates, significant others, or in off-campus social gatherings. Among the 378 close contacts, 116 (30.7%) received a positive test result. Percentages of positive test result rates were substantially higher among contacts with any unmasked exposure (114 of 352; 32.4%) than among those who had masked exposure only (two of 26; 7.7%) (aOR = 5.4, 95% CI = 1.5–36.5; p = 0.008) and for those who were unvaccinated (33.0%) or partially vaccinated (20.8%) compared with those who were fully vaccinated (none) (p = 0.007) (Table).

**TABLE:** Demographic characteristics, mask use, and number of exposure incidents of close contacts* of SARS-CoV-2–infected students, by SARS-CoV-2 test results — Saint Louis University, United States January–May 2021

Characteristic	SARS-CoV-2 test results, no. (%)			p value^†^
	All (n = 378)	Negative (n = 262)	Positive (n = 116)	
**Sex**				
Female	268 (70.9)	175 (66.8)	93 (80.2)	0.012
Male	110 (29.1)	87 (33.2)	23 (19.8)	
**Housing status**				
Off-campus	115 (30.4)	84 (32.1)	31 (26.7)	0.358
On-campus	263 (69.6)	178 (67.9)	85 (73.3)	
**Student level**				
Graduate	33 (8.7)	23 (8.8)	10 (8.6)	1.000
Undergraduate	345 (91.3)	239 (91.2)	106 (91.4)	
**Vaccination status** ^§^				
Fully vaccinated	18 (4.8)	18 (6.9)	0 (—)	0.007
Partially vaccinated	24 (6.3)	19 (7.3)	5 (4.3)	
Unvaccinated	336 (88.9)	225 (85.9)	111 (95.7)	
**Student athlete**				
No	330 (87.3)	228 (87.0)	102 (87.9)	0.939
Yes	48 (12.7)	34 (13.0)	14 (12.1)	
**Reserve Officers’ Training Corps member**				
No	376 (99.5)	261 (99.6)	115 (99.1)	1.000
Yes	2 (0.5)	1 (0.4)	1 (0.9)	
**Member of student fraternity or sorority**				
No	239 (63.2)	168 (64.1)	71 (61.2)	0.670
Yes	139 (36.8)	94 (35.9)	45 (38.8)	
**Health major with clinical responsibilities**				
No	294 (77.8)	201 (76.7)	93 (80.2)	0.541
Yes	84 (22.2)	61 (23.3)	23 (19.8)	
**Mask use**				
Masked exposure only	26 (6.9)	24 (9.2)	2 (1.7)	0.016
Any unmasked exposure	352 (93.1)	238 (90.8)	114 (98.3)	
**No. of exposure incidents,**^¶^ **median (IQR)**	1.0 (1.0, 2.0)	1.0 (1.0, 2.0)	2.0 (1.0, 3.0)	<0.001

**Abbreviation**: IQR = interquartile range.

* A close contact was defined as any unvaccinated person who spent a cumulative total of ≥15 minutes in one 24-hour period within 6 feet of a confirmed case of COVID-19 while that person was contagious, regardless of whether a mask was worn.

^†^ Determined by chi-square test for all comparisons except for number of exposure incidents (continuous variable), which was assessed using a *t* test.

^§^ Full vaccination was defined as having received either a single dose of Janssen (Johnson & Johnson) vaccine or the second dose of Moderna or Pfizer-BioNTech COVID-19 vaccine ≥14 days before the exposure. Partial vaccination was defined as receipt of a single dose of Janssen or the second dose of Moderna or Pfizer-BioNTech <14 days before the exposure, or receipt of 1 dose of Moderna or Pfizer-BioNTech COVID-19 vaccine. Being unvaccinated was defined as not having received a dose of any COVID-19 vaccine.

^¶^ Exposure incidents were defined as each single encounter in which a contact was within 6 feet of a student with COVID-19 for ≥15 minutes during a 24-hour period, regardless of location of the exposure.

In multivariate analyses, close contacts with unmasked exposure had higher adjusted odds of receiving a positive test result than did those with only masked exposure (aOR = 4.9; 95% CI = 1.4–31.1). Any additional exposure was associated with a 40.0% increase in adjusted odds of a positive test result (aOR = 1.4; 95% CI = 1.2–1.6); the median number of exposures was 2.0 for close contacts with positive test results and 1.0 for close contacts with negative test results. The two persons with only masked exposures who received positive test results were moved immediately to isolation upon receipt of test results. Neither of these two persons was linked to any additional COVID-19 cases, despite having nine close contacts between them and not having been placed into quarantine.

## Discussion

Close contacts with any unmasked exposure to persons with COVID-19 had significantly higher odds of receiving a positive SARS-CoV-2 test result compared with those who had only masked exposure. In addition, close contacts who had multiple exposures, whether masked or unmasked, had higher odds of a positive test result than did those with only a single exposure. The percentage of positive test results among close contacts in this study (30.7%) was similar to that observed in previous studies (approximately 31%) (*2*,*3*). Consistent with findings from studies in nonuniversity settings (*4*,*5*), the findings from this study reinforce that universal masking and having fewer encounters in close contact with persons with COVID-19 helps prevent further transmission in in-person university settings.

Two close contacts, who had only masked exposure and were permitted to forego quarantine because of the university’s modified protocol, received positive SARS-CoV-2 test results. Neither was linked to any additional COVID-19 cases (i.e., secondary transmission). For universities considering a similar approach, if masked unvaccinated close contacts are not required to quarantine, testing 5–7 days after exposure will be important because of the small risk for infection that could lead to secondary transmission if isolation is not implemented rapidly, especially in populations with low vaccination coverage. Modified quarantine, such as that implemented by this university, might be feasible on other university campuses or in other settings that have an internal contact tracing team and enforced testing protocols. For example, studies in K–12 school settings have found reduced SARS-CoV-2 transmission when masking is enforced even when 6 feet of physical distance cannot be maintained (*6*). Such modifications to quarantine policies might also have the potential to reduce the number of missed days from critical academic activities (e.g., clinical hours, flight hours) because of quarantine, which are days that are needed for degree completion and program accreditation. Modified quarantine, similar to that implemented by SLU, might also help minimize the negative psychosocial effects associated with quarantine (*7*). As of September 7, 2021, 1,014 universities have a COVID-19 vaccine requirement policy for students and employees (*8*).

The findings in this report are subject to at least five limitations. First, contact tracing data were self-reported, which could introduce social desirability or recall bias or inaccurate data regarding mask use. Second, the two students who received positive SARS-CoV-2 test results and reported only masked exposure might have had unmasked exposure to COVID-19 cases other than those under investigation, which could lead to underestimating the association between mask use and the percentage of positive test results. Third, most students were not vaccine-eligible until late spring, so this analysis included very few fully vaccinated students; therefore, vaccination status could not be included in the regression analysis because of low cell counts. Fourth, indoor versus outdoor exposure information and exposure time were collected but could not be included in the analysis because of a large amount of missing data. Finally, this study was conducted before the Delta variant became the dominant strain of SARS-CoV-2 in the United States, which could have an impact on these findings given that the Delta variant has increased transmissibility compared to previous variants.**

Wearing masks and having fewer encounters with persons with COVID-19 reduced the odds of transmission in a university setting. In addition, there was no evidence of secondary transmission from either of the two students with only masked exposure who received positive SARS-CoV-2 test results, and who, because of the modified protocol in place, were allowed to forego quarantine. Universities opening for in-person instruction could consider taking mask use into account when determining which unvaccinated close contacts require quarantine if enforced testing protocols are in place. CDC recommends that universal masking be adopted in indoor spaces for vaccinated and unvaccinated persons in areas with substantial or high transmission rates and that masks should be worn in indoor spaces in areas without substantial or high transmission rates if you are not fully vaccinated (*9*). In addition, CDC recommends COVID-19 vaccination for individuals aged ≥12 years.

**Summary**

**What is already known about this topic?**

During January–May 2021, Saint Louis University implemented a modified quarantine protocol that considered mask use when determining which close contacts required quarantine among an almost entirely unvaccinated student population.

**What is added by the report?**

Compared with only masked exposure, close contacts with any unmasked exposure had higher adjusted odds of a positive test result. Each additional exposure was associated with a 40% increase in odds of a positive test.

**What are the implications for public health practice?**

Universal masking and fewer encounters in close proximity to persons with COVID-19 can limit the spread of SARS-CoV-2 in university settings.

## Footnotes

* Quarantine is required for all unvaccinated close contacts who had any unmasked exposure. A close contact was defined as any unvaccinated person who spent a total of ≥15 minutes during a 24-hour period within 6 ft of a person with confirmed COVID-19 while that person was contagious, regardless of whether a mask was worn. The modified protocol allowed those with only masked exposure to forego quarantine.

† All symptomatic employees and students were offered COVID-19 testing through the Office of Employee Health or Student Health. Surveillance testing consisted of mandatory testing of all students living in on-campus housing before move-in for fall 2020 (August 2020) and spring 2021 (January 2021). Surveillance testing was optional for all students who lived off campus during fall and spring move-in. Throughout the fall 2020 and spring 2021 semesters, surveillance testing was conducted on a random sample of approximately 10% of students who lived in on-campus housing. In addition, student athletes and athletic staff members received surveillance testing per the National Collegiate Athletic Association (NCAA) guidelines: https://www.ncaa.org/sport-science-institute/covid-19-coronavirus

§ COVID-19 testing was conducted using Abbott BinaxNOW rapid antigen test and LabCorp molecular RT-PCR for all symptomatic persons collected via self-administered anterior nasal swab, or Clinical Reference Laboratory saliva-based molecular RT-PCR for all asymptomatic persons. Each symptomatic person was only counted once in the analyses, even though both an antigen and RT-PCR test were conducted.

¶ Logistic regression included masked status (any unmasked exposure versus only masked exposure) and number of exposure incidents (modeled as a continuous variable) with the persons with COVID-19 as predictors. These variables were chosen because of the strength of bivariate association with having a positive test result. The small number of close contacts with only masked exposures precluded more complex modeling; therefore, no demographic or other variables were included in the model. Linearity assumption was assessed by visual comparison of percentage of positive results by number of incidents. The model produced a Nagelkerke R-squared of 0.103 and a c-statistic of 0.654.

**
https://www.medrxiv.org/content/10.1101/2021.06.03.21258293v1

## References

[1] Losina E, Leifer V, Millham L, College campuses and COVID-19 mitigation: clinical and economic value. Ann Intern Med 2021;174:472–83. 10.7326/M20-6558 33347322 PMC7755069

[2] Denny TN, Andrews L, Bonsignori M, Implementation of a pooled surveillance testing program for asymptomatic SARS-CoV-2 infections on a college campus—Duke University, Durham, North Carolina, August 2–October 11, 2020. MMWR Morb Mortal Wkly Rep 2020;69:1743–7. 10.15585/mmwr.mm6946e1 33211678 PMC7676642

[3] Jones A, Fialkowski V, Prinzing L, Trites J, Kelso P, Levine M. Assessment of day-7 postexposure testing of asymptomatic contacts of COVID-19 patients to evaluate early release from quarantine—Vermont, May–November 2020. MMWR Morb Mortal Wkly Rep 2021;70:12–3. 10.15585/mmwr.mm7001a3 33411700 PMC7790157

[4] Cheng VC, Wong SC, Chuang VW, The role of community-wide wearing of face mask for control of coronavirus disease 2019 (COVID-19) epidemic due to SARS-CoV-2. J Infect 2020;81:107–14. 10.1016/j.jinf.2020.04.024 32335167 PMC7177146

[5] Pringle JC, Leikauskas J, Ransom-Kelley S, COVID-19 in a correctional facility employee following multiple brief exposures to persons with COVID-19—Vermont, July–August 2020. MMWR Morb Mortal Wkly Rep 2020;69:1569–70. 10.15585/mmwr.mm6943e1 33119564 PMC7640999

[6] Dawson P, Worrell MC, Malone S, ; CDC COVID-19 Surge Laboratory Group. Pilot investigation of SARS-CoV-2 secondary transmission in kindergarten through grade 12 schools implementing mitigation strategies—St. Louis County and City of Springfield, Missouri, December 2020. MMWR Morb Mortal Wkly Rep 2021;70:449–55. 10.15585/mmwr.mm7012e4 33764961 PMC7993558

[7] Kaparounaki CK, Patsali ME, Mousa DV, Papadopoulou EVK, Papadopoulou KKK, Fountoulakis KN. University students' mental health amidst the COVID-19 quarantine in Greece. Psych Res 2020 290:113111. 10.1016/j.psychres.2020.113111 32450416 PMC7236729

[8] Thomason A, O’Leary B. Here’s a list of colleges that require students or employees to be vaccinated against COVID-19. Washington, DC: Chronicles of Higher Education; 2021. Accessed September 7, 2021. https://www.chronicle.com/blogs/live-coronavirus-updates/heres-a-list-of-colleges-that-will-require-students-to-be-vaccinated-against-covid-19

[9] Brown CM, Vostok J, Johnson H, Outbreak of SARS-CoV-2 infections, including COVID-19 vaccine breakthrough infections, associated with large public gatherings—Barnstable County, Massachusetts, July 2021. MMWR Morb Mortal Wkly Rep 2021;70:1059–62. 10.15585/mmwr.mm7031e2 34351882 PMC8367314

